# Detection of Elevated Signaling Amino Acids in Human Diabetic Vitreous by Rapid Capillary Electrophoresis

**DOI:** 10.1155/2007/39765

**Published:** 2007-06-25

**Authors:** Miao-Jen Lu, Jose S. Pulido, Colin A. McCannel, Jose E. Pulido, R. Mark Hatfield, Robert F. Dundervill III, Scott A. Shippy

**Affiliations:** ^1^Department of Chemistry, University of Illinois at Chicago, 845 W Taylor St., Chicago, IL 60607, USA; ^2^Department of Ophthalmology, Mayo Clinic, 200 First St., S. W. Rochester, MN 55905, USA; ^3^Retina Consultants, Suite 301331 Laidley ST, Charleston, WV 25301, USA

## Abstract

Elevated glutamate is implicated in the pathology of PDR. The ability to rapidly assess the glutamate and amino acid content of vitreous provides a more complete picture of the chemical changes occurring at the diabetic retina and may lead to a better understanding of the pathology of PDR. Vitreous humor was collected following vitrectomies of patients with PDR and control conditions of macular hole or epiretinal membrane. A capillary electrophoresis method was developed to quantify glutamate and arginine. The analysis is relatively fast (<6 minutes) and utilizes a poly(ethylene)oxide and sodium dodecylsulfate run buffer. Both amino acid levels show significant increases in PDR patients versus controls and are comparable to other reports. The levels of vitreal glutamate vary inversely with the degree of observed hemorrhage. The results demonstrate a rapid method for assessment of a number of amino acids to characterize the chemical changes at the diabetic retina to better understand tissue changes and potentially identify new treatments.

## 1. INTRODUCTION

Signaling amino acids, including glutamate, *γ*-aminobutyric acid (GABA), and arginine are present in the vitreous and appear to play key roles in the pathology of variety eye disorders. Glutamate is one of the excitatory neurotransmitters in the retina. When it is present at high levels, it can over stimulate the N-methyl-D-aspartate type glutamate receptors on the ganglion cells of the retina and cause damage to the ganglion cells [1, 2]. GABA is an important inhibitory neurotransmitter in the retina and arginine, an essential amino acid, is also the precursor of the signaling molecule, nitric oxide (NO) [3]. The retinal vasculature is auto regulated and this appears to be related to nitric oxide [4]. In addition, there appears to be a relationship between nitric oxide and glutamate [5]. There are a few studies which have shown that in proliferative diabetic retinopathy (PDR), the levels of glutamate may be elevated [6]. Considering the fact that there are now glutamate antagonists that help in other diseases, it is important to do further studies to determine if the elevations of signaling amino acids like glutamate and arginine are elevated and therefore developing a rapid and better resolution method is necessary for vitreous analysis.

The vitreous is a chemically complex solution of carbohydrates, proteins, and small molecules. A number of analytical methods had been developed for vitreous analysis. Proteomic analysis via 2D gel electrophoresis with liquid chromatography electrospray tandem mass spectrometry (LC MS/MS) is the common strategy for proteins analysis in vitreous with or without diabetic retinopathy [7]. The use of enzyme-linked immunsorbet assays for preselected protein growth factors has been another approach [8, 9]. Small molecule analytes such as ascorbic acid [10], glutamate and GABA [11] have been evaluated with high-pressure liquid chromatography (HPLC) and HPLC coupled with electrochemical detection. Capillary electrophoresis-laser induced fluorescence (CE-LIF) is a powerful analytical tool for microliter to nanoliter volume biosample analysis that affords a number of advantages including faster separations, lower sample volume consumption, high resolution separations, and low limits of detection [12]. This technique has been used to evaluate inorganic ions in clinical vitreous samples [13–15]. We have previously described the use of CE for amino acid analysis of push-pull perfusates of the rat vitreous [16]. To date, there have been no reports of CE analysis of vitreous for amino acids separations that take advantage of the separation speed and resolution for understanding the chemical changes with diabetic retinopathy. In this study, we show further evidence that there is an elevation in glutamate and other signaling amino acids in PDR by using a rapid CE. Two separation conditions were explored in this study. The stability of amino acids from vitreous samples was also studied to improve monitoring of amino acid quantification.

## 2. EXPERIMENTAL

### 2.1. Chemicals

All chemicals were analytical grade and were purchased from Sigma (Sigma-Aldrich, St. Louis, MO, USA) unless otherwise noted. The labeling reagent 3-4-carboxybenzoyl quinoline-2-carboxaldehyde (CBQCA) was purchased from Molecular probes (Eugene, OR, USA). Sodium tetraborate, sodium phosphate, sodium dodecyl sulfate (SDS), *β*-cyclodextrin (*β*-CD), sodium hydroxide, sodium chloride (NaCl), potassium cyanide (KCN) were purchased from Acors (Fisher, Somerville, NJ, USA). Run buffer and acetic acid solution were freshly prepared in ultrafiltered, deionized water produced by US Filter purification system (Lowell, MA, USA).

### 2.2. Vitreous samples

This study was performed with institutional review board approvals at the participating institutions; the University of Illinois Chicago, Chicago, IL, Saint Francis Hospital, Charleston, WV, and the Mayo Clinic, Rochester, MN. The characteristics of the patient population is summarized in Table 1. Patients undergoing vitrectomy for idiopathic epiretinal membranes or idiopathic macular holes and who had no other ocular problems served as controls (*n* = 25) to compare to those who had vitrectomy for PDR with hemorrhage classified from none to severe (*n* = 18). At the start of the vitrectomy, before the infusion was started, a pure vitreous sample was removed with the vitrector and 100 *μ*L volume of vitreous was pipetted into centrifuge tube that contained 100 *μ*L 1 M acetic acid (pH 2 ∼ 3) to deactivate endogenous proteases. Vitreous samples of patients were delivered to University of Illinois at Chicago by overnight courier.

Analysis of 2 *μ*L aliquots began with derivatization of vitreous amino acids with 2 *μ*L 20 mM CBQCA in the presence of 2 *μ*L 10 mM cyanide. Because the pH value of vitreous samples (pH 2-3) was not compatible with the derivatization reaction, the cyanide solution was prepared in 250 mM phosphate buffer with pH 10 to adjust the pH. The reaction mixture of vitreous sample and CBQCA was allowed to react for 2 hours at room temperature prior to CE-LIF analysis similar to previously published results [16].

### 2.3. Running condition for MEKC method

A separation method for low-flow push-pull perfusates of the rat vitreous had been described previously [16]. These conditions were tried in this study. The run buffer consisted of 20 mM sodium tetraborate (pH 9.3), 20 mM sodium chloride, 45 mM sodium dodecyl sulfate (SDS), and 55 mM *β*-cyclodextrin (*β*-CD). In order to explore a faster separation for these studies, the capillary was reduced to 40 cm (30 cm effective length). All separations were performed at 17 kV applied voltage.

### 2.4. Running conditions for MEKC-PEO method 

An alternative separation method was developed using 0.6% linear polymer, polyethylene oxide (PEO; MW = 600 kDa) added to the separation buffer. PEO was prepared in 20 mM sodium tetraborate (pH 9.3), 20 mM sodium chloride, and 75 mM sodium dodecyl sulfate (SDS) run buffer. Before introducing sample into capillary, the capillary has been rinsed with 50 mM sodium tetraborate buffer (pH 9.3) to fill the capillary with a low viscosity solution to allow gravimetric injection. The separation was performed with the inlet end of the capillary in the PEO buffer at 16 kV applied voltage.

### 2.5. Instrumentation of CE-LIF

The CE-LIF system used in these studies has been described previously [16]. Briefly, the system was built in-house with a high voltage power supply (Spellman, Hauppage, NY, USA), a Zetalif Lif detector (Picometrics, Paris, France), and an argon ion laser (Coherent, Santa Clara, CA, USA) at 488 nm. Buffer vials were placed in a plexiglass box to isolate high voltages. The LabView programming environment (National Instruments, Austin, TX) was used with a custom program for instrument control and data acquisition. All CE analyses were performed at room temperature with a 40 cm × 360 *μ*m O.D. × 50 *μ*m I.D. (30 cm effective length) fused-silica capillaries (BioTAQ, Gaithersburg, MD, USA). In this work, gravimetric sample injection was used with a 30 cm height difference for 5 seconds.

### 2.6. Data analysis

The collected data were analyzed with Microsoft Excel and Class Eleganza CE station software V5.5 (Ayer Rajah Industrial Estate, Singapore). Amino acid quantification was performed with calibration curves. In order to confirm the validity of amino acids concentration determinations from calibration curves, the method of standard additions was used randomly with a number of vitreous samples for comparison. An F-test was performed to evaluate the equivalence of control and PDR data set variances for the appropriate application of a nonpaired student's *t*-test to determine statistical significance. A one-way ANOVA (*P* < .05) was performed on amino acid data for groups of low/moderate/high levels of vitreal hemorrhage.

## 3. RESULTS AND DISCUSSIONS

### 3.1. New separation method for chemically complex human vitreous samples

The goal of this project was to explore the combination of MEKC with a highly viscous buffer, in this case a PEO-containing solution. The analysis of small molecule amino acids is compared with a previous method in terms of its speed and resolution. The optimized separation conditions are then used for the clinical analysis of a number of vitreous samples.

#### 3.1.1. Use of developed method for rat vitreous perfusate-MEKC method

**Figure 1:**
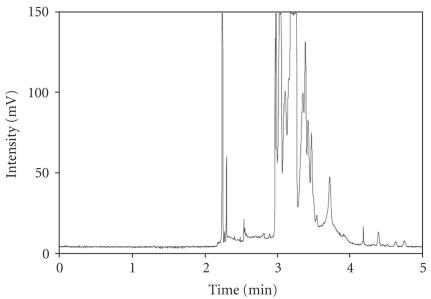
Electropherogram of MEKC method. MEKC method was developed for rat vitreous perfusate. The following is the separation condition for MEKC method. Capillary used for separation is 40 cm in total length and 30 cm in effective length. Composition of the run buffer is 20 mM sodium tetraborate, 20 mM sodium chloride, 45 mM sodium dodecyl sulfate (SDS), and 55 mM *β*-cyclodextrane (*β*-CD). Sample injection is 5 seconds by hydrodynamic injection and the separation was conducted at 17 kV. Note the poor separation of the peaks following three minutes.

In the beginning of this work, the separation condition that had been described previously [16] was evaluated the use for human vitreous samples. This separation condition is the MEKC method. The developed separation method was used for amino acids analysis for rat vitreous perfusates. In order to do relatively faster separations, we decreased the capillary length down to 40 cm compared to previous work to decrease the analysis time by 50% (<5 minutes) (see Figure 1). Because rat vitreous perfusates and human vitreous samples are quite different especially since there was addition of acetic acid into human vitreous samples to deactivate proteases, the separation based on the MEKC method applied to human vitreous analysis did not separate the peaks as well as in the rat vitreous perfusates.

#### 3.1.2. Newly developed MEKC-PEO method for amino acid analysis of human vitreous

*Development of MEKC-PEO method*

**Figure 2:**
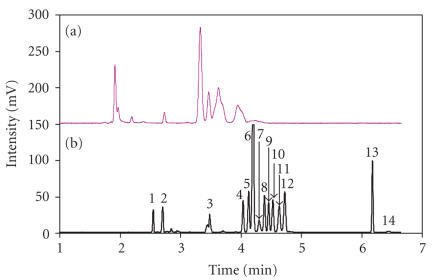
Separation of amino acid standards in MEKC-PEO method, (a) 0 mM SDS and (b) 75 mM SDS. Separation buffer composed by 0.6% polyethylene oxide (PEO), 20 mM sodium tetraborate, and 20 mM sodium chloride. Before introducing sample into capillary, capillary was filled out with 50 mM sodium tetraborate buffer and following by sample injection through hydrodynamic injection. After sample injection, separation buffer was introduced into capillary by electro-osmotic flow (EOF) and separation was conducted at 16 kV. Electropherogram of standard amino acid mixture. Identification is shown in (b) as follows: 1: Arginine, 2: Histamine, 3: Lysine, 4: Methionine, 5: Histidine, 6: Phenylalanine, 7: Valine, 8: Serine, GABA, 9: Tyrosine, 10: Alanine, 11: Taurine, 12: Glycine, 13: Glutamate, 14: Aspartate.

**Figure 3:**
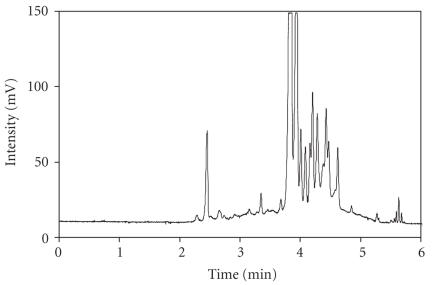
Representative electropherogram of a vitreous sample. Separation condition is the same as Figure 2(b).

Polyethylene oxide (PEO) had been used for small molecules analysis of body fluids, but it is usually used as a stacking matrix in CE separation. Because the PEO would increase the viscosity of the separation buffer, this affects the efficiency of hydrodynamic sample injection. The capillary was filled with 50 mM borate buffer as the leading buffer to allow sample injection as well as regenerate high and reproducible electroosmotic flow. A solution of fifteen amino acids was used to determine if PEO as a sieving matrix could help in improving the resolution of amino acids. Figure 2(a) shows that only 7 peaks were resolved out of 15 amino acids so using PEO as a sieving mechanism was not sufficient to separate the amino acids. Therefore we used PEO coupled with MEKC method to improve the resolution. Sodium dodecyl sulfate (SDS) is the most common surfactant used for micellar electrokinetic chromatography (MEKC). SDS concentrations in the 25–100 mM range were evaluated for optimization. The 75 mM SDS addition gave the best separation and peak shapes. This allowed successful separation of 14 peaks out of 15 amino acids (see Figure 2(b)). Applying the optimized condition to vitreous sample analysis showed that with SDS and PEO, a better resolution was achieved within 6 minutes compared to prior methods used in previous studies from other research groups (see Figure 3).

From the results, we hypothesize that in our MEKC-PEO method, the micelles interact with PEO to improve the resolution by increasing the difference of mobility of neutral small molecules, such as amino acids. In addition, the PEO molecules would absorb to the capillary wall thereby preventing proteins from adhering. This would allow better separation of complex biosamples which contain a variety of proteins.

The MEKC-PEO method does not provide the ability to separate _D_- and _L_- serine as the MEKC method also employs the chiral selector, *β*-cyclodextrin. Common to both methods is the fact that GABA is either not found [16] with the MEKC buffer or coelutes with Ser with the MEKC-PEO buffer. Improved detector sensitivity will be needed to quantify GABA. However, the amino acid taurine is easily separated by the MEKC-PEO method in contrast to the MEKC method.

When PEO is used as a stacking matrix, a relatively long (∼30 minutes) separation time is required due to the concentration of the amino acids from large plugs of sample solution. In this case PEO is playing a role as a sieving matrix, and allowed the analysis time to be shortened to less than 6 minutes.

### 3.2. Concerns about human vitreous samples preparation

#### 3.2.1. Hyaluronic acid effect

Hyaluronic acid (HA) is the most important cause of the viscosity of the vitreous. The viscosity may play an important role in separation therefore, to determine the role of HA in the separation of vitreous amino acids, hyluronidase (HAase) was used to degrade HA in the vitreous samples. Vitreous samples were incubated with HAase for 1 to 3 hours with different volume ratios ranging from 1 : 1 to 1 : 5 (HAase: vitreous; *μ*L), and then evaluated by CE-LIF analysis. We found that there was no significant change in peak heights and although there was a slight increase in retention time (data not shown). Our results indicate that the presence of hyaluronic acid does not affect the ability to separate amino acids.

#### 3.2.2. Vitreous sample stability study over time

**Figure 4:**
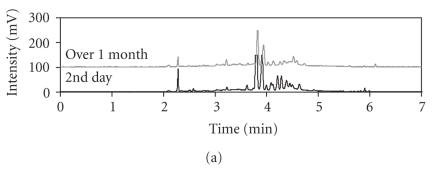

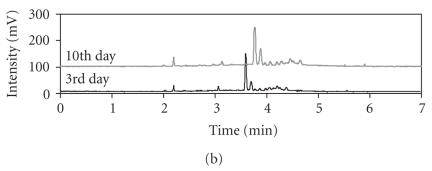
Electropherograms comparing human vitreous stability over time. The separation conditions are the same as Figure 2(b). Electropherograms (a) compares the traces found at day 2 following collection to one performed at one month following collection and shows a large dissimilarity in peaks; (b) electropherograms performed at 3 days and 10 days since vitreous sample collection shows similar peaks. Electropherograms (a) and (b) are from different patients. The stability of human vitreous sample begins to degrade after two weeks since the time of collection.

Another concern was the stability of the vitreous samples especially since samples in our situation are shipped from several locations. It is well known that vitreous sample contains a variety of proteolytic enzymes that would break down proteins to small peptides and free amino acids. The activity of those proteolytic enzymes might cause an overestimation of amino acid levels. In general, the enzyme activity is pH dependent and most of the proteolytic activity is degraded when the pH is below 4 so 1 M acetic acid solution was introduced to vitreous samples. In addition to clarify the vitreous sample's stability over time vitreous samples were evaluated when they arrived, at two weeks and at one month by CE-LIF analysis. The electropherograms of the vitreous samples at initial determination and at two weeks begin to show a degradation that is enhanced by one month. Figures 4(a) and 4(b) are representative electropherograms from different patients. Figure 4(a) shows that the stability of vitreous sample over one month is decreased while in Figure 4(b) the vitreous sample had been analyzed at three days and ten days following collection and showed minor changes. Therefore, amino acid analysis of vitreous sample should be done within 1 week for quantitative accuracy.

### 3.3. Investigation of PDR and ERM/MH

**Figure 5:**
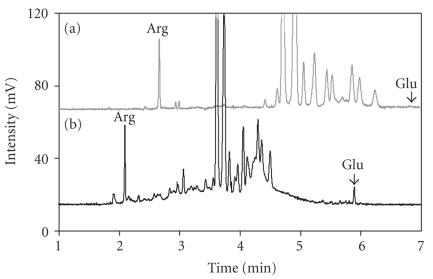
Electropherogram comparison for (a) MH and (b) PDR in MEKC-PEO method. Patient with MH has simpler peak pattern than PDR and MH group had better resolution than PDR.

Based upon a comparison of the electropherograms of samples from the PDR and control groups, the peak patterns between PDR and ERM/MH group appeared markedly different (see Figure 5). The peak pattern of PDR group is more complex than ERM/MH group. There were more peaks in the PDR electropherograms and the resolution in the PDR group was not as good as ERM/MH group. This suggests that the vitreous samples from patients with PDR contain a greater amount and an increase in the types of proteins and small peptides.

**Figure 6:**
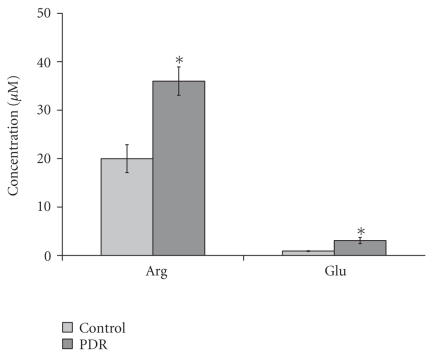
Human vitreous Arg and Glu levels in control and PDR groups. Each bar represents the mean ± SEM of numbers of subjects. There were significant differences between Arg and Glu levels of control and PDR groups (*P < .05).

To quantitate the differences in the signaling amino acids, peak heights averaging was used to determine the concentrations. The summary of the Arg and Glu levels of the ERM/MH and PDR groups are shown in Figure 6. Glutamate levels for the groups are: PDR = 3.08 ± 0.65 *μ*M (*n* = 18) and ERM/MH = 0.91 ± 0.08 *μ*M (*n* = 24) (*P* < .005). The results here confirm and add to the few prior studies showing an elevation of glutamate with PDR [6, 7]. Arginine levels were also significantly higher: PDR = 35.99 ± 2.93 *μ*M (*n* = 18) and ERM/MH = 19.98 ± 2.89 *μ*M (*n* = 24) (*P* < .005).

**Figure 7:**
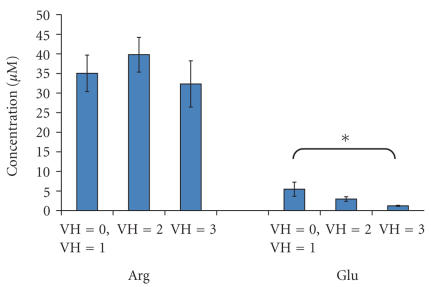
Bar graph of the Arg and Glu levels in PDR group according to extent of vitreal hemorrhage. The gradations of hemorrhage were classified as follows: 0: none, +1: mild, +2: moderate, and +3: severe. Significance is determined by one-way ANOVA *P* < .05.

There are differences in the degrees of vitreal hemorrhage among the patients in this study that may cause variances in the observed amino acid levels. The concentration of blood is generally higher than what is measured here in the vitreous such that leakage from blood could cause an increase in the levels of glutamate seen in the vitreous [17]. To account for this, the PDR group was broken into three levels of vitreous hemorrhage. In Figure 7 there is a bar graph of the means ± SEM of the Arg and Glu concentrations in each group. There is a significant decrease in the Glu level with increasing severity of vitreous hemorrhage (*P* < .05) while there is no difference in the observed Arg concentration. These data are somewhat surprising but demonstrate the power of our quantification method. The changes observed in Glu, but not Arg suggest that there are likely neurochemical changes at the retina prior to observable hemorrhage that may be leading the damage at the diabetic retina.

In conclusion, we describe the use of micelle coupling with PEO as a sieving matrix for small molecules analysis in bodily fluid. This new MEKC-PEO method allowed us to do the amino acid analysis of vitreous with faster separation and better resolution. In addition, we have found that addition of acetic acid to vitreous samples allows determination of amino acids for up to two weeks following collection. Finally, we confirm that PDR causes a statistically significant elevation in glutamate levels and also show that arginine levels are elevated.

## Figures and Tables

**Table 1:** Patient characteristics.

	Control (ERM/MH)	PDR

Sex (M/F)	11/14	9/9
Type 1/Type 2/no diabetes	0/4/21	4/14
Age (mean ± SEM, years)	67 ± 2	53 ± 3
Duration of diabetes (mean ± SEM, years)	10 ± 3	19 ± 2
Blood glucose (mean ± SEM, mg/dl)	113 ± 10^a^	151 ± 21^b^
Vitreous hemorrhage (−, +1, +2, +3)^c^	25, 0, 0, 0	1, 4, 7, 6
Traction detachment (−, +1, +2, +3)^d^	25, 0, 0, 0	14, 3, 0, 1

^a^ *n* = 21, ^b^ *n* = 16, ^c^ ‘−’ = none, +1 = mild, +2 = moderate, +3 = severe, ^d^ ‘−’ = none, +1 = mild-focal area, +2 = moderate-area less than 2dd, +3 = severe.
